# The Human FcγRII (CD32) Family of Leukocyte FcR in Health and Disease

**DOI:** 10.3389/fimmu.2019.00464

**Published:** 2019-03-19

**Authors:** Jessica C. Anania, Alicia M. Chenoweth, Bruce D. Wines, P. Mark Hogarth

**Affiliations:** ^1^Centre for Biomedical Research, Burnet Institute, Melbourne, VIC, Australia; ^2^Department of Immunology and Pathology, Central Clinical School, Monash University, Melbourne, VIC, Australia; ^3^Department of Pathology, The University of Melbourne, Melbourne, VIC, Australia

**Keywords:** Fc receptor, FcγR, inflammation, infection, autoimmunity, cancer, mAb therapeutics

## Abstract

FcγRs have been the focus of extensive research due to their key role linking innate and humoral immunity and their implication in both inflammatory and infectious disease. Within the human FcγR family FcγRII (activatory FcγRIIa and FcγRIIc, and inhibitory FcγRIIb) are unique in their ability to signal independent of the common γ chain. Through improved understanding of the structure of these receptors and how this affects their function we may be able to better understand how to target FcγR specific immune activation or inhibition, which will facilitate in the development of therapeutic monoclonal antibodies in patients where FcγRII activity may be desirable for efficacy. This review is focused on roles of the human FcγRII family members and their link to immunoregulation in healthy individuals and infection, autoimmunity and cancer.

## Introduction

Fc receptors are, by definition, receptors for the Fc portion of immunoglobulins (Ig). These have been traditionally viewed primarily as cell surface receptors for Ig and whose interaction drives a surprisingly diverse range of responses mostly within the immune system or related to the physiology of antibodies in immunity.

Receptors for IgM, IgA, IgG, and IgE have been defined over the last 40 years with the majority of research focused on the receptors found on leukocytes. These receptors induce or regulate leukocyte effector functions during the course of immune responses. It is noteworthy, and also beyond the scope of this review, that a limited number and type of Fc receptors are also expressed on cells outside the immune system where they affect or participate in physiology of antibody function.

In humans, the largest grouping of Fc receptors is the “leukocyte Fc receptors” expressed primarily on effector cells. Their ectodomains bind ligand, the IgG antibody Fc region, and belong to the Ig-superfamily. They include the high affinity IgE receptor FcεRI and the distantly related IgA receptor FcαRI, but the largest group are the IgG receptors or the FcγRs which themselves comprise several groups—FcγRI, the high affinity IgG receptor, the FcγRII family (FcγRIIA, FcγRIIB, FcγRIIC), and the FcγRIII family (1, 2).

## The Human FcγRII (CD32) Family of Leukocyte FcR

### General Comments

The human FcγRII family (also known as CD32 in the Cluster of Differentiation nomenclature) consists of a family of primarily cell membrane receptor proteins. They are encoded by the mRNA splice variants of three highly related genes—*FCGR2A, FCGR2B*, and *FCGR2C*, which arose by recombination of the *FCGR2A* and *FCGR2B* genes (3).

All members of the FcγRII family are integral membrane glycoproteins and contain conserved extracellular domains, exhibiting an overall 85% amino acid identity (3, 4). The high degree of amino acid and DNA identity has posed challenges in the analysis of receptor function using monoclonal antibody or nucleic acid based methods. Thus, some caution should be exercised when analyzing literature or interpreting experimental data. The encoded products of the three genes are low-affinity receptors that are defined practically as interacting poorly with monomeric IgG, i.e., micromolar affinity (5, 6), but when arrayed on the cell surface, they avidly bind multivalent complexes of IgG, e.g., immune complexes.

The FcγRIIA (also FcγRIIC) and FcγRIIB proteins have opposing cellular functions. FcγRIIA proteins are activating-type Fc receptors. In contrast, FcγRIIB is a key immune checkpoint that modulates the action of activating-type Fc receptors and the antigen receptor of B cells. When expressed, the FcγRIIC proteins retain the activating function of the cytoplasmic tail of FcγRIIA and the binding specificity of FcγRIIB ectodomains.

The focus of this review is the FcγRII family and their actions as receptors for immunoglobulins. It should be noted that FcγRIIA also acts as a receptor for pentraxins, a product of innate immunity that is important in infection and inflammation and which has been recently reviewed elsewhere (7). Since much of the biology of the Fc receptors has been determined in the mouse, it is noteworthy that the human and mouse FcR families differ significantly, with FcγRIIB being the only FcγRII forms in the mouse. Also, although the human and mouse FcγRIIB homologs are highly conserved, there are differences in their splice variants in the two species (see below). Importantly, cellular expression can also vary between humans and mice.

Human FcγRII gene polymorphism, mRNA splicing, and copy number variation (CNV) further diversifies the potential biological consequences of IgG interactions with the FcγRII receptor proteins. These properties and roles of each group of FcγRII proteins are reviewed in detail in the following sections.

## Properties oF FcγRIIA

### Molecular Structure

**Figure 1:**
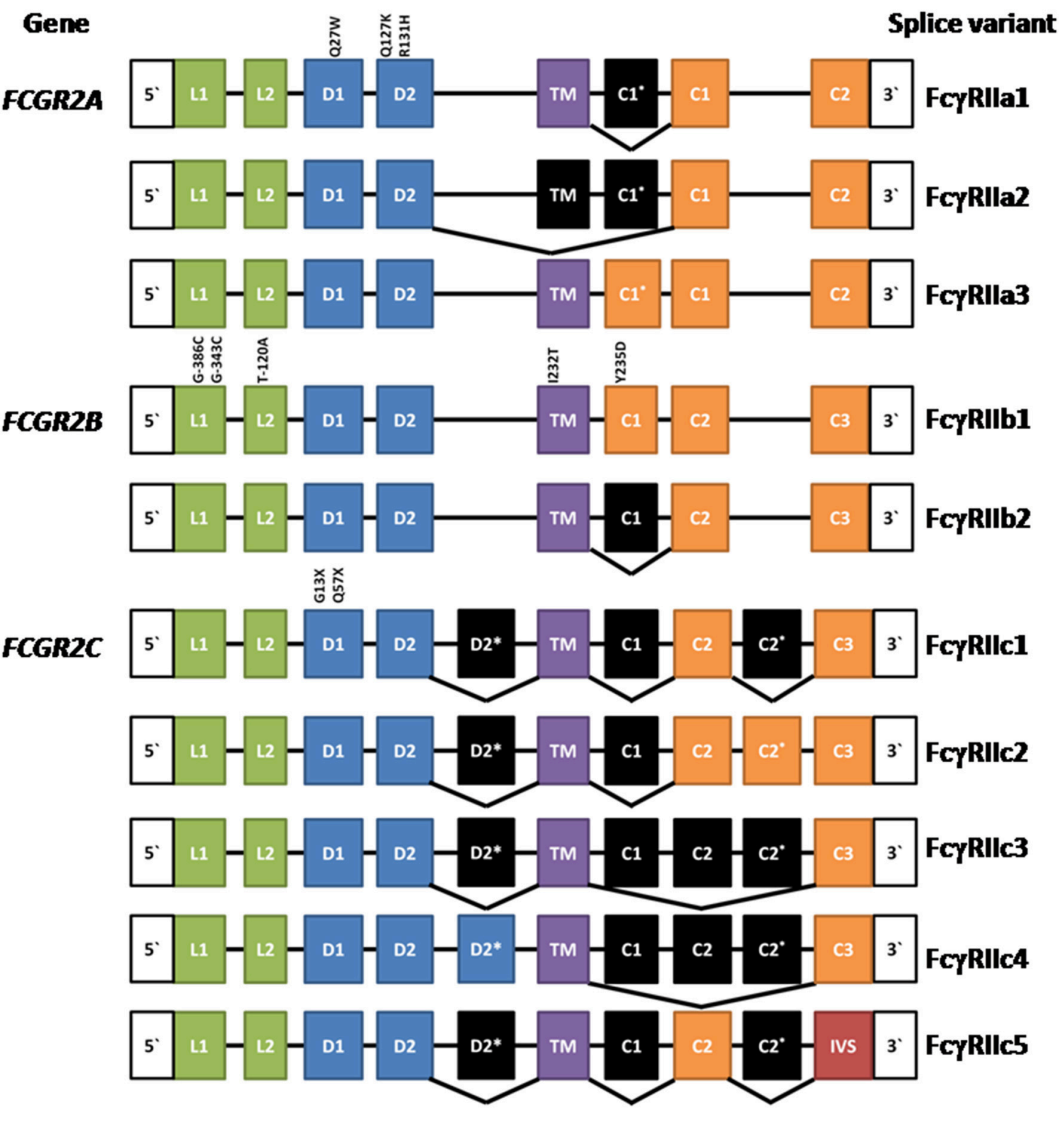
Composition of *FCGR2A, FCGR2B*, and *FCGR2C* and their splice variants. Leader (L), ectodomain (D), transmembrane (TM) cytoplasmic tail (c), and intervening sequence (IVS). Expressed exons are illustrated in color, while spliced exons (selectively expressed) are represented in black. The location and position number of amino acids affected by well characterized polymorphisms are shown above the exons except for the *FCGR2B* leader exons where the nucleotide positions are given. See text for references.

The human FcγRIIA proteins were originally defined by cross-species gene cloning (8). They are encoded by the *FCGR2A* gene (Figure 1) and are comprised of eight exons; two encoding the 5‘ UTR, and leader sequence and the N-terminus of the mature protein; one exon for each of the two Ig-like domains of the extracellular region; one exon for the transmembrane domain; and three exons encoding the cytoplasmic tail and 3′ UTR (3). Three mRNA transcripts, two of which encode membrane proteins, arise by alternative splicing of the mRNA (Figure 1).

The most extensively characterized form is the canonical 40 kDa integral membrane protein, FcγRIIA1, that contains all but the first (C1^*^) cytoplasmic sequence (3, 4, 8–10). A second, but relatively rare, membrane form has been recently described (11, 12). FcγRIIA3 is identical in sequence to the canonical FcγRIIA1, with the notable exception of a 19-amino acid insert in its cytoplasmic tail, arising from the inclusion of the C1^*^ exon which was believed previously to be a vestigial or cryptic exon (4). This insertion is highly homologous (18/19-amino acids) to the insertion present in the cytoplasmic tail of inhibitory FcγRIIB1 (11–13). mRNA splicing that successfully gives rise to FcγRIIA3 is associated with an *FCGR2A*^c.7421871A>G^ SNP that creates a splice acceptor site, which greatly increases the inclusion of the C1^*^ exon (11).

An unusual mRNA has been reported that lacks the transmembrane exon resulting in a potentially secreted 32 kDa polypeptide (14). This FcγRIIA2 form is not extensively characterized and its physiology is uncertain. However, it raises the possibility that naturally occurring soluble forms may act as modulators of immune complex-induced activation and inflammation and it is noteworthy that recombinant soluble FcγRIIA inhibits immune complex-induced activation of inflammatory cells *in vitro* and *in vivo* (9).

### Cellular Expression

The FcγRIIA proteins are unique to primates (15, 16). FcγRIIA1 is the most widespread and abundant of all FcγR, present on Langerhans cells, platelets and all leukocytes, with the exception of most lymphocytes (Table 1) (1, 16, 17). FcγRIIA3 is expressed by neutrophils and monocytes (11) and FcγRIIA2 mRNA is present in platelets, megakaryocytes, and Langerhans cells (14). The levels of FcγRIIA expression are influenced by cytokine exposure. Interferon (IFN)-γ, interleukin (IL)-3, IL-6, IFN-γ, C5a, prostaglandin-E (PGE), and dexamethasone increase expression, but IL-4, tumor necrosis factor (TNF)-α, and TNF-β reduce expression (18–21). There are also reports of FcγRII induction on CD4 and CD8 T cells upon mitogen or TCR stimulation. Both FcγRIIA and FcγRIIB are reported to be expressed on activated CD4 T cells (22, 23).

**Table 1:** Leukocyte Expression of FcγRII forms.

**Cell type**	**FcγRIIA**	**FcγRIIB**	**FcγRIIC^**a**^**
T cells	i*^b^*	i*^b^*	?
B cells	—	+++	+
NK cells	—	—^c^	+
Macrophages	+++	++	?
Monocytes	+++	+	?
Neutrophils	+++	+	?
Eosinophils	++	•	•
Basophils	++	+++	—
Mast cells	++	—*^d^*	—
Platelets	++	—	—

*+++ High, ++ Moderate, + Low, or — No expression. • no data*.

a *Expressed only in ~20% of humans*;

b *Expression induced in some T cell subpopulations*;

c *Expressed as a result of promoter modification related to FcγRIIC allelism*.

d *Conflicting results*.

### FcγRIIA Signaling ITAM Activation vs. ITAM Inhibition

Like other activating-type immunoreceptors, FcγRIIA and FcγRIIC signal via the Immunoreceptor Tyrosine-based Activation Motif (ITAM) pathway (24–26) with a major structural difference. In the case of all other activating-type immunoreceptors—which includes the antigen receptors as well as the activating type FcR, e.g., FcεRI, FcγRIIIA—the ligand binding chain and the signaling subunits are encoded in separate polypeptides e.g., FcγRIIIA and the common FcR-γ chain dimer. The assembly of a functional signaling complex requires their non-covalent association (17). However, in the case of FcγRIIA and FcγRIIC, the ITAM is present in its own IgG binding chain. Furthermore, the FcγRIIA ITAM is unusual in that it does not fit the canonical ITAM consensus sequence and includes three additional aspartic residues (Table 2), although how this affects FcγRII function remains unknown (13). ITAM signaling is essential for FcγRIIA-dependent phagocytosis and the induction of cytokine secretion induced by its aggregation by immune complexes. Such high stoichiometry aggregation of receptors results in receptor-associated src family kinase, particularly Fyn (27), mediated phosphorylation of the two tyrosines of the ITAM and the recruitment of Syk and the propagation of activatory signaling pathways. In human FcγRIIA transgenic mice, Fyn deficiency is protective in models of FcγR dependent nephritis and arthritis, indicating a pivotal pro-inflammatory role for Fyn kinase in ITAM signaling (27).

**Table 2:** Sequence comparison of ITAMs of activating type FcγR.

**Receptor ITAM**	**Consensus^**a**^**
FcR-γ chain	**Y**TG**L** STRN———QET **Y**ET**L**
FcγRIIA and Fcγ IIC	**Y**MT**L** NPRAPTDDDKNI **Y**LT**L**

a *Bold letters in FcRγ chain and FcγRIIA sequences indicate the critical Tyr and Leu residues of the ITAM consensus motif YxxL/I (6–12) YxxL*.

Since the original characterization of the activating role of ITAM pathway was described, it is now apparent that ITAMs can under certain circumstances mediate inhibitory or modulating function termed ITAMi (inhibitory ITAM) (28, 29). Under conditions of low stoichiometric interaction, the receptor-associated src family kinase Lyn phosphorylates only one of the two tyrosine residues (mono-tyrosine phosphorylation) within the ITAM, with two juxtaposed receptors presenting mono-phosphylated-ITAMs to recruit the two SH2 domains of the SH2-domain containing protein tyrosine phosphatase 1 (SHP-1). This interaction is not dissimilar to SHP1 binding via its dual SH2 domains to inhibitory immunoreceptors with dual ITIMs (30). Then Lyn phosphorylation of Tyr^536^ of SHP-1 positively regulates SHP-1 phosphatase activity resulting in the inhibition of cell activation (27). Animal studies suggest that the ITAMi effect ameliorates pathological inflammatory responses and may also be important in controlling “baseline” receptor activation. This ITAMi effect is not unique to the unusual FcγRIIA ITAM (29) as it has been also described for FcαRI (31, 32) and FcγRIIIA (33), both of which signal through the common FcR-γ chain dimer which contains canonical ITAMs.

### Cellular Responses

FcγRIIA aggregation by IgG cross-linking initiates a variety of effector responses, depending on cellular expression which is affected by the local cytokine environment, and cross-talk between other FcR and TLR (34, 35). Internalization via both endocytosis and phagocytosis can be mediated by FcγRIIA in cell lines, i.e., ts20 (36, 37), COS-1 (38), U937 (39) as well as in primary human cells i.e., neutrophils (40, 41), monocytes (40), platelets (40, 42), and macrophages (43). FcγR phagocytosis requires ITAM activation, which also initiates the ubiquitin conjugation system. Conversely, endocytosis is dependent only on ubiquitination and clathrin, not ITAM phosphorylation (36, 37).

The internalization of antigen: antibody immune complexes by FcγR on antigen presenting cells (especially dendritic cells) is an important part of antigen presentation for the development of effective immune responses. This process also increases the efficiency of T cell activation particularly in response to low concentrations of antigen (44). The role of human FcγR in antigen presentation is well documented in *in vitro* systems and it appears that all FcγR are important at some level (45–47). However, more recent analyses have shown FcγRIIA is the major receptor in the development of so-called “vaccinal effects” of monoclonal antibody therapy in cancer. It appears that the therapeutic antibodies targeting cancer cells can induce a long lasting protective response beyond the acute therapeutic phase of the therapy (48).

FcγRIIA1 activates neutrophils and other myeloid effector cells for direct killing of IgG-opsonized target cells including tumor cells and virus-infected cells (49). Also, FcγRIIA binding of IgG immune complexes triggers granulocytes to release inflammatory mediators such as prostaglandins, lysosomal enzymes, and reactive oxygen species, as well as cytokines including IFNγ, TNFα, IL-1, and IL-6 (50, 51). The FcγRIIA3 splice variant form is an even more potent activator of human neutrophils than FcγRIIA1, and is responsible for some severe adverse reactions to immunoglobulin replacement therapy (11). The mechanistic basis of this potency relates to its longer retention time in the cell membrane and the consequential enhanced ITAM signaling (12). Whilst this enhanced potency may present a risk factor for hypersensitivity to immunoglobulin replacement therapy, it may provide some benefit for protection against infection.

The limited number of studies of FcγRII expression of human T cells suggest FcγRII crosslinking on TCR-stimulated CD4 T cells enhances proliferation and cytokine secretion, suggesting an activating function of FcγRIIA (22, 23). The nature of FcγRIIA expression on CD4 T cells is not straightforward nor completely characterized. Purified CD4 T cells when stimulated with anti-CD3/CD28 induced surface expression of FcγRII on 10% of cells and intracellular expression in 50%. In contrast, unstimulated cells express little FcγRII (23). Imaging of FcγRII-expressing CD4 T cells sorted from unstimulated normal peripheral blood mononuclear cells, or those from HIV-1^+^ individuals shows cells displaying punctate FcγRIIA staining (23) or discrete patches of B cell membrane. These B cell membrane patches include FcγRIIB and CD19 markers (52), consistent with possible trogocytosis by the activated T cell from the B cell. Similarly, FcγRIIIA is also expressed on activated CD4 T cells, and this expression appears to be both intrinsic upon cell activation and acquired by trogocytosis of APC membrane (53).

FcγRIIA plays an important role in the normal physiology of platelet activation, adhesion, and aggregation following vessel injury (54). More recent studies indicate FcγRIIA associates with glycoprotein (GP) Ib-IX-V on platelets and can thereby be indirectly stimulated by von Willebrand factor (VWF) or after stimulation of G-protein-coupled receptors (GPCRs) (54). Interestingly, FcγRIIA signaling on platelets is regulated by proteolytic cleavage of the cytoplasmic tail, or “de-ITAM-ising” (55).

## Properties OF FcγRIIB

### Molecular Structure

Initially, FcγRIIB was discovered in the mouse by protein sequence and molecular cloning analyses (56, 57) and the human *FCGR2B* gene was then isolated by cross species hybridization. Human *FCGR*2*B* has similar structure to human *FCGR2A*, being comprised of eight exons. The two major forms of FcγRIIB—FcγRIIB1 and FcγRIIB2 (Figure 1)—arise from mRNA splicing which results in the inclusion or exclusion of the C1 exon sequence in FcγRIIB1 and FcγRIIB2 isoforms, respectively (3, 4). The inclusion of the C1 exon sequence in the FcγRIIB1 results in tethering to the membrane of B cells, whereas its absence from FcγRIIB2 allows rapid internalization of the receptor in myeloid cells. Both forms contain the Immunoreceptor Tyrosine-based Inhibitory Motif (ITIM) in their cytoplasmic tails. The extracellular domains are 95% identical to the two domains of FcγRIIA and almost completely identical to the FcγRIIC (3, 8, 17). Although the focus of this review is the human FcγRII, it should be noted that mouse FcγRIIB comprises three splice variants FcγRIIB1, FcγRIIB1′, and FcγRIIB2, with the predicated amino acid sequences of the latter two corresponding to the human FcγRIIB1 and FcγRIIB2 variants. Any functional differences between the two mouse FcγRIIB1 and FcγRIIB1′ forms are unknown (58). There are also amino acid sequence differences between human FcγRIIB1 and mouse FcγRIIB1/1′ and the functional consequences of these are also unknown.

### Cellular Expression

As indicated in “General Comments” above, the analysis of expression of human FcγRIIB protein has been historically difficult because of the extremely high sequence conservation of the extracellular domains of FcγRIIB, FcγRIIA, and FcγRIIC and lack of specific monoclonal antibody probes. The high degree of DNA sequence conservation has also confounded analysis. Much of the early literature has relied on either PCRs or interpretation of data using antibodies that are cross-reactive with, or specific for, FcγRIIA or a combination of these methods and reagents. The relatively recent development of such FcγRIIA/C and FcγRIIB specific antibodies (59–61) has now helped to clarify expression patterns, but there are still differences reported between groups using these reagents. Some caution should still be exercised in analysis of the historic literature. Furthermore, cell expression patterns of FcγRIIB in mouse myeloid derived cells is substantially different to human FcγRIIB, thus additional caution is advised in interpreting the data. Nonetheless, it is clear that FcγRIIB (FcγRIIB1) is highly expressed by B cells, and its mRNA has also been identified at lower levels on monocytes (Table 1) (62). The levels of FcγRIIB expression are influenced by cytokine exposure. Cytokines such as IL-10, IL-6, and dexamethasone increase expression of FcγRIIB, while TNF-α, C5a and IFN-γ inhibit expression (18–20).

FcγRIIB (FcγRIIB2) is highly expressed on basophils and at low levels on monocytes (63). Expression on other granulocytes is somewhat complex and controversial. The differences in reported expression of FcγRIIB on mast cells may reflect technological limitations or differences in tissue origin of the cells under investigation. Intestinal and cord blood derived mast cells have been reported as expressing FcγRIIB on the basis of mRNA expression (64). In one study using human leukocyte reconstituted mice and a FcγRIIB specific polyclonal antibody, FcγRIIB protein was detected (65). However, skin mast cells lack FcγRIIB surface expression (66) and using a FcγRIIB specific mAb, peripheral blood derived mast cells do not express FcγRIIB (A. Chenoweth personal communication). Neutrophils either lack (60) or express very low levels of FcγRIIB (59), and the FcγRIIB-specific mAb 2B6 does not usually stain NK cells (60). However, in that proportion (~20%) of the population where FcγRIIC is expressed, NK staining by FcγRIIB antibodies might be expected as FcγRIIC EC domain is identical to FcγRIIB. A further complication is that FcγRIIC CNV affects control elements of the *FCGR2B* gene permitting FcγRIIB expression in NK cells (67) (see FcγRIIC below).

One of the more interesting features of FcγRIIB is its presence on non-leukocyte cells including airway smooth muscle (68) and liver sinusoidal endothelial cells (69). Its abundance in liver, in the mouse, accounting for three quarters of the total body expression, appears to provide a large sink for the removal in IgG immune complexes, which has been exploited in therapeutic monoclonal antibodies whose Fc portions have been engineered for high affinity binding to FcγRIIB (70, 71). This appears to be a “stand alone” function of FcγRIIB where small immune complexes are internalized without risk of pro-inflammatory activation.

### FcγRIIB Modulation of Immunity

FcγRIIB was the first immune “checkpoint” defined (72), with mouse studies showing a pivotal role in controlling autoreactive germinal center B cell activation and survival in mice with dysfunction resulting in loss of tolerance and autoimmunity (73, 74). Mice with humanized immune systems reconstituted with stems cells homozygous for the dysfunctional FcγRIIB Thr^232^ allele develop autoantibodies with specificities characteristic of lupus and human rheumatoid arthritis (75). This critical action of its ITIM in controlling the ITAM activation pathway is extensively reviewed elsewhere (25, 76). The ratio of activating vs. inhibitory receptors is a key factor in determining the cellular threshold for cell activation and resulting immune response (18, 77). An ITIM, consensus amino acid sequence YXXL (where X represents any amino acid), is found in the cytoplasmic domains of both FcγRIIB1 and FcγRIIB2. The co-engagement of FcγRIIB with an activating type receptor such as FcγRIIA or the B cell antigen receptor (25) modulates their ITAM-mediated activation signal. FcγRIIB expression on innate effector cells modulates cell activation mediated by activating FcγRs, including dendritic cell maturation and antigen presentation. FcγRIIB also regulates signaling from varied innate cell receptors including TLRs and complement receptors, reviewed in Bournazos et al. (34) and Espeli et al. (78).

Much of the detail in understanding of the ITIM:ITAM system of immune cell modulation has been derived from FcγRIIB1 ITIM-mediated regulation of the B cell receptor (BCR) signaling in mouse B cells. Conventional FcγRIIB-mediated inhibition requires ligand-dependant co-engagement/aggregation of ITAM-containing receptors (79, 80). The FcγRIIB ITIM modulation targets the two major ITAM driven pathways—ITAM tyrosine phosphorylation, and the generation of phospholipid mediators, e.g., Phosphatidylinositol (3,4,5)-trisphosphate (PIP3). Briefly, src kinases such as Lyn kinase, which participate in the phosphorylation of the ITAM of the ligand-clustered activating receptors, also phosphorylate the FcγRIIB ITIM of the co-aggregated inhibitory receptor. Notably, FcγRIIB1 has been reported to be phosphorylated by Lyn and Blk, whereas FcγRIIB2 solely by Blk (81).

The phosphorylated-ITIM of FcγRIIB recruits the inositol phosphatases SHIP1 and SHIP2, as is extensively reviewed in Getahun and Cambier (25). The preferential recruitment of SHIP, over SHP1 and SHP2, to the phosphorylated FcγRIIB cytoplasmic domain is determined by the SHIP SH2 domain's affinity for the pITIM (82). Notably studies of SHIP recruitment to the cytoplasmic domain of mouse FcγRIIB1 found phosphorylation of Tyr^326^, outside the ITIM, bound the SH2 domain of the adaptor Grb2 which bridged and stabilized the FcγRIIB:SHIP complex (83). Human FcγRIIB lacks an equivalent tyrosine, and has a small adjacent deletion. It fails to recruit Grb2 but still recruits SHIP1 that modulates BCR-induced Ca mobilization (84). SHIP dephosphorylates phosphatidylinositol species, with the predominant *in vivo* substrate being phosphatidylinositol 3,4,5-trisphosphate and ultimately recruits p62 Dok to form a highly active membrane localized enzymatic complex. This inhibits the Ras activation pathway, decreases MAP kinase activation and reduced PLCγ function leads to less activation of PKC. SHIP-dependent ITIM inhibition of the MAP kinase pathway, together with the anti-apoptotic kinase Akt can thereby affect cellular proliferation and survival (25).

The same mechanisms defined for BCR regulation are applicable to human and mouse myeloid cells, where many observations have been confirmed, particularly for FcγRIIB2 regulation of FcεRI (25, 76). Overall FcγRIIB1 and FcγRIIB2 signaling pathways are similar, however their principal functional difference lies in their localization in the cell membrane. The C1 insertion (85) of FcγRIIB1 prolongs membrane retention, whereas FcγRIIB2 is rapidly internalized. The equivalent C1^*^ sequence in FcγRIIA3 also alters membrane localization (see above).

An ITIM independent mechanism of B cell regulation by FcγRIIB has been reported wherein FcγRIIB, by binding antigen bound IgG, co-aggregates with the BCR and prevents the membrane organization of BCR and CD19 (86, 87). In another mode of regulation of the adaptive humoral response, FcγRIIB has been reported to be expressed on plasma cells and binding IgG immune complexes and trigger apoptosis (88). Studies have also identified other mechanisms of FcgRIIB modulation of the IgE receptor and the BCR the existence of which in human cells has not been determined. Mouse bone marrow derived mast cells, which differ phenotypically from human mast cells, showed an unconventional FcγRIIB ITIM-dependent regulation of the high affinity IgE receptor, FcεRI, where intracellular mediated co-aggregation of FcεRI with FcγRIIB occurs independently of the FcγRIIB ectodomain binding to antigen complexed IgG (89).

### Cellular Responses

The specific effects of FcγRIIB signaling are dependent on the context of the co-engaged activating receptors and the cell type. In B cells, FcγRIIB1 inhibition of the BCR is a critical immune checkpoint for regulating antibody production (25, 90). The powerful nature of this immune checkpoint is evident from studies in clinical, genetic, and animal models that show that altering the balance between ITIM modulation and ITAM activation is central to the pathogenesis and severity of disease (91).

As humoral immune responses develop, circulating antigen:antibody complexes simultaneously engage the antigen-specific BCR via the antigen of the complex and FcγRIIB via the Fc region, thereby modulating antigen receptor signaling. In FcγRIIB1, the C1 insertion impairs endocytosis, increasing the interaction time between FcγRIIB1, and the BCR. The C1 insert, irrespective of its position in the cytoplasmic tail, tethers the receptor to the cytoskeleton and so prevents the receptor localizing to coated pits and so disrupting endocytosis (92, 93). A di-leucine motif within the FcγRIIB ITIM sequence is also required for endocytosis (93, 94). Thus, the C1 insert confers cytoskeletal tethering and membrane retention which counter other cytoplasmic tail sequences including the di-leucine residues that would otherwise promote endocytosis.

FcγRIIB2 has also been studied in B cells in experimental systems where it also co-engages the BCR and regulates its function. FcγRIIB2 lacks the cytoplasmic C1 insertion and is rapidly internalized. A rare Tyr^235^Asp polymorphism occurs within the unique membrane-tethering 19-amino acid insertion of FcγRIIB1. FcγRIIB1-Asp^235^ binding of mouse IgG1 was slightly lower in comparison to the Tyr^235^ variant of FcγRIIB1, as was mIgG1 anti-CD3 induced T cell mitogenesis (95, 96). FcγRIIB1-Asp^235^ retained the capacity to form caps and was effective in down-regulating increases in calcium upon cross-linking by serum IgG (95).

This prolonged surface expression of actively signaling FcγRIIB1 may also be important for the elimination by apoptosis of self-reactive B cells during somatic hyper-mutation (97). Thus, FcγRIIB1 constrains the selective antigen specificity of the humoral immune system and directs the B cell production toward an appropriate antibody repertoire.

FcγRIIB is upregulated after antigen stimulation via immune complexes on follicular DCs (FDCs) (98). FDCs retain immune complexes and recycle them periodically to their plasma membrane, a process believed to be important in development of B cell immune cell memory (99). The presentation of immune complexes by activated FDCs expressing FcγRIIB provides antigens to B cells in a highly immunogenic form by multimerising the antigens, thus extensively crosslinking multiple BCRs, minimizing B cell FcγRIIB ITIM-mediated inhibition and providing co-stimulatory signals (100).

The functional response of a cell that expresses both ITAM-bearing receptors and FcγRIIB can be altered by their expression levels. Basophils express activatory FcεRI and FcγRIIA, as well as FcγRIIB, which can inhibit IgE-induced responses (101, 102). This balance can be altered by IL-3 which upregulates expression of both FcγR, but more strongly enhances FcγRIIB2 expression (101). Under normal physiologic conditions it is believed that FcγRIIA co-aggregation may, by providing activated Lyn, aid FcγRIIB inhibitory function (102).

Monocyte-derived dendritic cells (moDCs) that were treated with IFNγ to upregulate their activating FcγRs (FcγRI and FcγRIIA) had increased IgG-mediated cellular maturation, while moDCs treated with anti-inflammatory concentrations of soluble monomeric IgG (IVIg) to increase FcγRIIB expression had decreased cellular maturation (18). Similarly, monocytes with increased expression of activating FcγRs over FcγRIIB as induced by IFNγ or TNFα had enhanced IgG-triggered cytokine production, while monocytes with enhanced FcγRIIB expression by IL-4 and IL-10 prevented IgG-triggered cytokine production (103). Furthermore, FcγRIIB^−/−^ mouse macrophages developed robust inflammatory responses after exposure to subthreshold concentrations of immune complexes that failed to induce responses in FcγRIIB-expressing cells, demonstrating a role of FcγRIIB in setting a “threshold” for cellular activation (104).

## Properties OF FcγRIIC

### Molecular Structure

The expression of the membrane *FCGR2C* is complex. It is subject to a polymorphism (Gln^13^STOP) wherein ~80% of the population do not express functional FcγRIIC proteins and also CNV, which in turn impacts expression of the *FCGR2B* gene as described above (67, 105). The *FCGR2C* gene arose by recombination between *FCGR2B* and *FCGR2A*. The functional transmembrane FcγRIIC protein encoded by this gene is an activating receptor wherein the extracellular domains are derived from and are identical to FcγRIIB (exons 1–4), but the transmembrane and cytoplasmic tail are derived from the activating type ITAM-containing FcγRIIA (exons 5–8).

Multiple mRNA splice variants of FcγRIIC have been identified (Figure 1), though their physiology is unclear. Interestingly, some FcγRIIC-Gln^13^ individuals still lack FcγRIIC expression due to alternative splicing that gives rise to multiple non-functional forms (67). Additionally, the *FCGR2C* locus shows CNV, which may contribute to variation in gene expression, at the transcript and/or protein level, also impacting other FcγRII expression and function (67, 106).

### Expression and Cellular Responses

In individuals expressing the activatory FcγRIIC, it has been most extensively studied on NK cells (Table 1). NK cells expressing FcγRIIC had increased levels of ADCC upon receptor cross-linking, causing mediator release and lysis of target cells (67, 105–108). Although not extensively studied, it appears that FcγRIIC is also expressed on CD19+ B Cells. Its co-ligation with the BCR caused enhanced BCR signaling and B cell function, relative to FcγRIIB ITIM-dependent negative regulation in the absence of FcγRIIC. This FcγRIIC expression on B cells is associated with systemic lupus erythematosus (SLE) in humans, possibly related to the altered or unbalanced ITAM/ITIM signaling (108).

Interestingly, multiple other SNPs, 114945036, rs138747765, and rs78603008, have been significantly associated with FcγRIIA or FcγRIIC mRNA expression in B cells in European populations (109). However, protein expression data is not yet available.

## Structural Basis OF FcγRII Interaction With IgG

Human FcγRs have distinct binding specificities and affinities for the four IgG subclasses (2). The determination of affinity and IgG subclass specificity has relied on a wide range of methods mostly based on the binding of immune complexes to cell-expressed FcγR. More sensitive methods have used recombinant ectodomains and monomeric IgG using highly sensitive cell free systems such as SPR (5, 6, 110). A survey of the literature on the measurement of specificity and affinity of these receptors shows some variation in the methods used and the values calculated. Even the application of more sophisticated methods such as SPR show some degree of variation from group to group. Notwithstanding the variations and limitation in analyses of the interactions, it is clear that the FcγRII family (FcγRIIA, FcγRIIB, and FcγRIIC), are sensors of immune complexes and as such, interact poorly with uncomplexed monomeric IgG (1 μM affinity) but avidly bind immune complexes (5, 6, 15, 110).

There is general agreement that all FcγRII, indeed all FcγR, bind human IgG1 and IgG3 but there are significant differences in the interaction with IgG2 and IgG4 (Table 3). The allelic His^131^ form of human FcγRIIA is the only receptor which avidly binds human IgG2 complexes, while FcγRIIA-Arg^131^ binds IgG2 poorly (Table 3). However, it is possible that under circumstances of high local concentrations of opsonizing antibodies that binding interactions occur with FcγRIIA-Arg^131^ though whether there is a functional outcome is unknown (6, 111).

**Table 3:** Relative binding of human IgG by FcγR expressed on the cell surface.

**Human FcγR**	**Human IgG Subclass**			
	**IgG1**	**IgG2**	**IgG3**	**IgG4**
FcγRIIA His^131^	+++	++	++++	–
FcγRIIA Arg^131^	++	±	++++	±
FcγRIIB	+	–	+++	+
FcγRIIC	+	–	+++	+

In contrast, FcγRIIB binds IgG4 but not IgG2 and moreover, binds IgG1 and IgG3 an approximately 10-fold lower affinity than the activating FcγRIIA. This is consistent with its powerful physiological inhibitory function as IgG binding affinities equal to or higher than the activating receptors might otherwise prevent pro-inflammatory responses that are necessary in resisting infection. Not surprisingly, FcγRIIC has the same IgG binding properties at FcγRIIB (6).

Other factors that affect interactions between IgG and the FcγRII are the size of the IgG immune complex (112), the distribution of epitopes (111, 113), the geometry of the Fc in the complex, and receptor localization in membrane domains (114) which may also influence the avidity of immune complex binding. The state of the cell expressing the receptor (115) can also influence interaction with IgG. FcγRIIA function may be modified by “inside-out signaling” whereby external stimuli such as granulocyte-macrophage colony-stimulating factor (GM-CSF), IL-5, and IL-3 in eosinophils (116) and N-formylmethionyl-leucyl-phenylalanine (fLMP) in neutrophils increase receptor avidity (117). The mechanism for this FcγRIIA “activation” is unknown but could involve receptor dimer forms (5, 115, 117, 118). This inside-out signaling has also been identified for the high affinity IgG receptor, FcγRI, where it is associated with cytoskeletal-dependent clustering of receptors (119).

**Figure 2:**
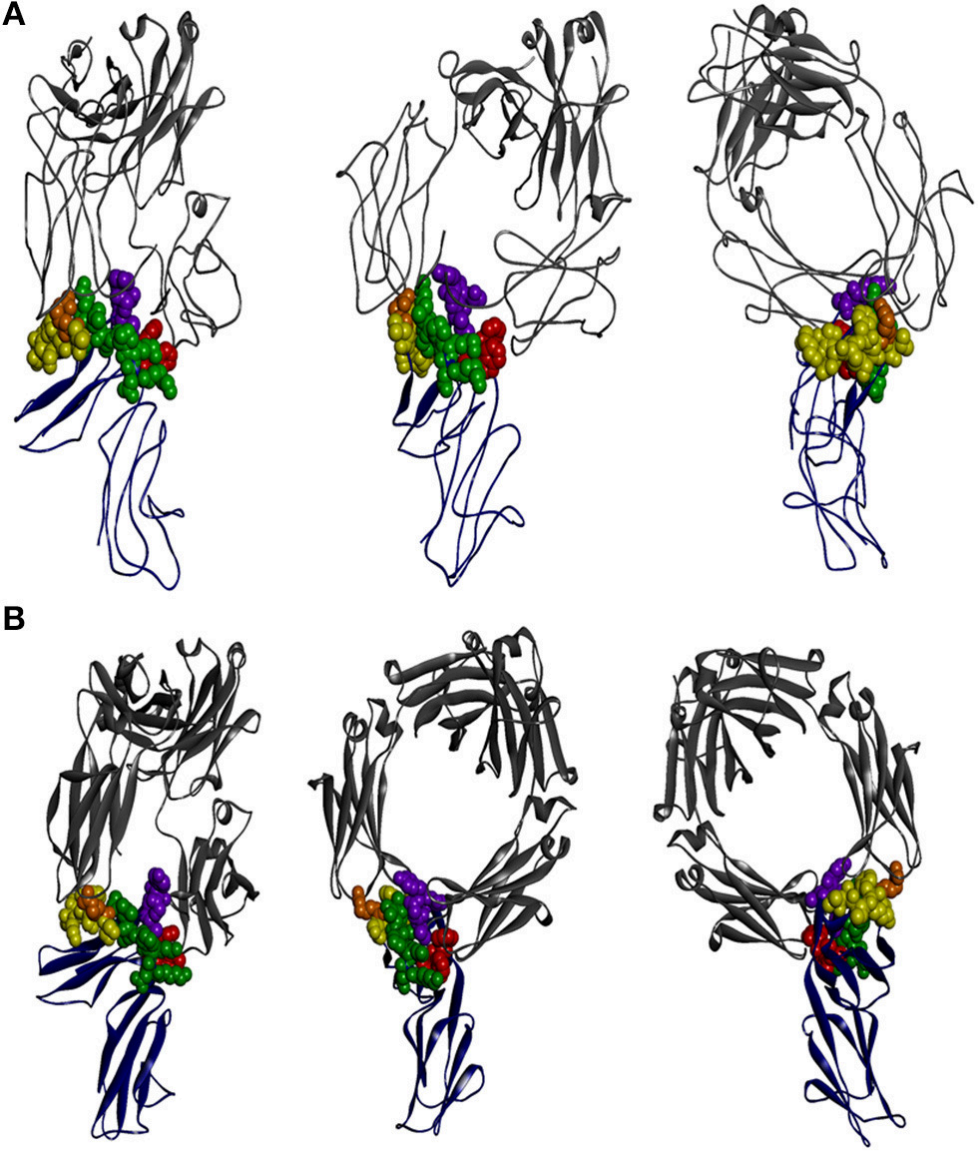
The interaction of IgG-Fc with FcγRIIA and FcγRIIB is similar. The perspectives shown are of two ectodomains of the **(A)** FcγRIIA [adapted from 3RY6 (61)] and **(B)** FcγRIIB [adapted from 3WJJ (123)] (shown in dark blue) in complex with IgG-Fc (shown in gray). The structural components of the receptor contributing to IgG binding are the two tryptophan residues that form the Trp sandwich (red), the BC loop (green), the C′E strand (yellow), and the FG loop (purple), with the “high/low resonder” polymorphic residue His^131^Arg highlighted (orange).

X-ray crystallographic structural data is available for all FcγR but only in complex with the native or mutated IgG1 (61, 120–122). It is clear that the interaction of FcγRIIA and FcγRIIB with IgG1 is asymmetric. The “bent” FcγR extracellular region of one FcγR molecule inserting between, and making contacts with, both IgG1 H-chain Fcs, as is also the case with other FcγR (Figure 2) (2, 124). The key conclusion from these studies is that the principal contact regions of the FcγRIIA and FcγRIIB are similar and occur predominantly within the second domain BC loop, C strand, C′E loop, and the FG loop, with a contribution of the interdomain linker. The BC loop and the interdomain linker provide the two critical tryptophan residues, conserved in all FcγR, that sandwich the Pro^331^ of the IgG1 CH2 FG loop.

The lower hinge of IgG has a dominant role in determining the specificity of FcγR interactions. In the case of IgG1, the lower hinge residues, Pro^233^Leu^234^Leu^235^Gly^236^Gly^237^, of both H-chains form extensive contacts with FcγRIIA (61). Interestingly, this region is quite different in IgG2 (Pro,Val,Ala,Gly) and suggests that the IgG2 interaction with FcγRIIA may be quite distinct at the atomic level but as yet no structure of IgG2 in complex with FcγRIIA is known. Nonetheless, the IgG1:FcγRIIA complex structure suggests that the preferential IgG2 binding by FcγRIIA-H^131^ over FcγRIIA-R^131^–the “high/low responder” polymorphism (125)—may be explained structurally by the smaller histidine side chain more readily accommodating interaction with the Fc adjacent to the lower hinge compared to the longer arginine side chain (61).

The structural basis for the effect of the rare Gln^127^Lys polymorphism that also affects IgG2 binding is interesting (126). The Lys^127^ does not appear to make contact with the IgG1 Fc and sits adjacent to the binding region, so that the effect on Fc binding is presumably indirect. This indicates a possible selective pressure for IgG2 binding by this receptor (126).

## Roles OF FcγRII in Health and Disease

The balance between activation and inhibitory signaling is important in the control of healthy antibody dependant responses and disturbance to this balance can have adverse, but in some cases positive, consequences to health.

Genetic polymorphism studies of human *FCGR2* genes have helped to establish roles of FcγRII proteins in several autoimmune diseases and in resistance or susceptibility to infectious diseases (Table 4). *In vivo* mechanistic studies in experimental animal models, including transgenic and gene replacement systems, have also been helpful in establishing specific protective or deleterious roles of FcγRII in infectious disease, inflammation, autoimmunity, and cancer and have been reviewed extensively elsewhere (139–143).

**Table 4:** Function or clinical association of polymorphic residues of FcγRII.

**Receptor**	**Polymorphism**	**Function/clinical association**	**Reference**
FcγRIIA	Gln27Trp (rs9427397, rs9427398)	Impaired calcium mobilization and MAP kinase phosphorylation; associated with CVID	(127)
	Gln127Lys	Gln^127^ interferes with the interaction of adjacent receptor residues with IgG2	(126)
	His131Arg (rs1801274)	His^131^ able to bind IgG2; both forms associated with autoimmune disease; allograft rejection and mAb cancer treatment outcomes	(128, 129)
	c.7421871A>G	Permits alternative splicing of the C1* exon resulting in expression of “hyperactive” FcγRIIA3. Risk factor for IVIg anaphylaxis.	(11, 12)
	Hypomethylation	Increased susceptibility genes for Kawasaki disease and IVIg resistance	(130)
FcγRIIB	Promoter haplotype (rs3219018, rs34701572)	Deregulated FcγRIIB expression may contribute to pathogenesis	(59, 131)
	Ile232Thr (rs1050501)	Thr^232^ allele does not partition to lipid rafts and is associated with impaired regulation of ITAM signaling, predisposing to SLE but protective for malaria	(132–137)
	Tyr235Asp	Asp^235^ has reduced binding, internalization and signaling	(95, 96)
FcγRIIC	Gln13stop	Commonly referred to as the ORF/Stop polymorphism, determines functional expression of receptor, may contribute to autoimmune disease	(105, 106)
	Gln57stop (rs1801274)	Unknown mechanism, associated with autoimmune disease and vaccine efficacy for HIV	(106, 138)

### Infection

The *in vivo* roles of the FcγRII receptor family in humans have been derived by extrapolation of animal studies and by genetic studies of human populations. The FcγRIIA high/low-responder polymorphism influences susceptibility to infections, as FcγRIIA-Arg^131^ has poor IgG2 binding (144, 145). Individuals expressing FcγRIIA-His^131^ are more resistant to infection by *Streptococcus pneumonia, Haemophilus influenza*, and *Neisseria meningitides*. This is potentially due to more avid binding of IgG2 by FcγRIIA-His^131^ over FcγRIIA-Arg^131^, consequently resulting in more efficient effector responses such as uptake by phagocytes, induction of degranulation and elastase release by granulocytes *in vivo* (144, 146, 147).

FcγRs do not function in isolation under physiological conditions *in vivo* and it is notable that co-operation between Toll-like receptors (TLRs) and FcγRs is an important feature of effective pathogen elimination (148). TLRs are often co-expressed with FcγRIIA and co-engagement results in enhanced functional responses of these individual receptors, e.g., enhanced TNFα, IL-23, and IL-1β release by DCs (35, 149, 150).

The role of FcγR in HIV is complex and apparently conflicting data may reflect different aspects of HIV infection and clinical outcomes. In a small study of immunocompetent patients who had undergone successful and early antiretroviral treatment, who expressed FcγRIIA-His^131^, and had a IgG2 response to a gp120 vaccine regime, there was a partial control of viral replication during interruption of anti-retroviral therapy (151). However, analysis of the Vax004 gp120 vaccine trial found no evidence of association of FcγRIIA polymorphism with protection against HIV infection, although this was an unsuccessful vaccine trial overall (152). HIV studies have emphasized the protective role of NK cell FcγRIIIA in antibody dependent cellular cytotoxicity. However, recent studies have found a potent role for FcγRIIA in the protective functions of macrophages and neutrophils, which are abundant effectors at the mucosal sites of HIV acquisition (153). HIV co-infections generate an even more complex clinical picture. FcγRIIA-His^131^ homozygous individuals are more susceptible to developing AIDs-related pneumonia, and have an increased risk of placental malaria in HIV-infected women (154) and other perinatal infections (155, 156).

While few resting CD4 T cells express FcγRIIA, these cells are highly relevant to HIV research. Resting CD4 T cells latently infected with HIV are an important target in strategies to eliminate HIV in anti-retroviral therapy (ART) patients, as these quiescent cells provide safe harbor for “silent” virus that, upon reactivation, causes viral recrudescence within weeks of treatment interruption. FcγRIIA was reported as a surface marker of this key quiescent population in ART patients (157) but other studies found no enrichment of HIV proviral DNA by sorting CD4 T cells based on FcγRIIA expression (52, 158). Rather than on resting CD4 T cells, FcγRII expression was mostly on activated CD4 cells associated with transcriptionally active virus (159). Furthermore, another study sorted a CD4^+^ population that apparently expressed FcγRIIB, not FcγRIIA. However, these FcγRIIB^+^ cells derived from contaminating B cells, occurring as T-B cell doublets, and also from single CD4 T cells, with a punctate staining pattern that included other B cell markers, and was suggestive of trogocytosis rather than intrinsic CD4 T cell expression (52). These studies indicate some of the technical challenges that can accompany determining FcγR expression.

Though the numbers are small there is suggestive evidence that polymorphism in the *FCGR2C* locus, in particular *FCGR2C*-126 C>T SNP was associated with a protective anti-HIV vaccination response. In the RV144 vaccine trial, individuals homozygous for *FCGR2C*-126C/C had an estimated vaccine efficacy of 15% whereas individuals homozygous for the *FCGR2C*-126T/T or heterozygous−126 C/T had an estimated vaccine efficacy of 91% (138). Whether this association relates to effector function via a functional FcγRIIC protein or is due to linkage to another effector system encoded in this chromosomal region is uncertain (109).

FcγRs also have an established role in antibody-dependent enhancement (ADE) of dengue virus (DENV) infection. Immune complexes of DENV opsonized with non- or sub-neutralizing levels of antibodies interact with FcγRs on monocytes, macrophages, and dendritic cells, led to increased uptake, viral replication, and more severe infection (160). In keeping with its modulating role, FcγRIIB inhibits ADE in experimental systems (161). Indeed, while FcγRIIA facilitates DENV entry, mutation of the ITAM to an ITIM significantly inhibited ADE, and conversely, replacing the inhibitory motif in FcγRIIB with an ITAM, conferred ADE capacity (162).

The hypo-functional FcγRIIB-Thr^232^ variant is enriched in populations from malaria endemic areas. This suggests that reduced FcγRIIB modulation of responses and a consequential enhancement of B cell and inflammatory cell activation confers a survival advantage in these populations (132, 163). Indeed, enhanced activatory FcR responses including increased phagocytic capacity and TNF production by innate cells and enhanced B cell responses is evident by elevated malaria specific antibody titers (164).

Interestingly, the FcγRIIB-Thr^232^ polymorphism has been shown to confer increased phagocytosis of antibody opsonized bacteria by monocyte-derived macrophages (132). Models suggest FcγRIIB is integral for the balance between efficient pathogen clearance and the prevention of the cytokine-mediated effects of sepsis (163). In geographic areas where there is less infectious disease pressure, FcγRIIB-Thr^232^ is associated with susceptibility to autoimmunity.

### FcγR in Autoimmunity

Imbalance between inhibitory and activatory FcγR functions predisposes individuals to pro-inflammatory autoimmune disease. FcγRIIA activation induces the production of pro-inflammatory cytokines, including IFN and TNFα, which are active in the promotion of inflammation, systemic lupus erythematosus (SLE), Kawasaki disease (KD), Grave's disease, and Rheumatoid Arthritis (RA) (35, 165–167).

The FcγRIIA-His^131^ allelic form is associated with other autoimmune diseases, including Guillain-Barré syndrome, ulcerative colitis and KD, possibly due to increased inflammatory cell activation via IgG2 (168–170).The FcγRIIA-Arg^131^ allelic form is associated with susceptibility to SLE, angina pectoris, acute coronary syndrome (ACS), myasthenia gravis, and RA (171–174). This may be related to the impaired ability of FcγRIIA-Arg^131^ to process and recycle IgG2, causing the release of pro-inflammatory cytokines, aggravating disease (175, 176).

Other FcγRIIA polymorphisms, although less well characterized, are associated with inflammatory diseases. Recently a glutamine/tryptophan polymorphism at position 27 (Gln^27^Trp) has been identified, where homozygous individuals were over represented in CVID (127). No difference in expression was observed and FcγRIIA-Trp^27^ had modest impairment of calcium mobilization and MAP kinase phosphorylation *in vitro* (127).

Epigenetic modifications of *FCGR2A* such as hypomethylation have also been described in CVID patients, particularly at the promoter CpG site cg24422489 (130, 169). This increased susceptibility for KD and resistance to Ig replacement therapy, with significant hypomethylation of FcγRIIA in patients with acute KD and coronary artery lesions (130, 169, 177).

The recently described rare intronic A>G SNP that controls expression of the splice variant FcγRIIA3 occurs in <1% of healthy subjects (11, 12). However, it is associated with KD, immune thrombocytopenia (ITP), and CVID (11). Furthermore, severe adverse reactions in response to immunoglobulin replacement therapy occurred in patients expressing FcγRIIA3 and neutrophil activation (mediator and elastase release) was enhanced. Increased signaling by FcγRIIA3 was due to its altered membrane localization and longer membrane retention time (11, 12). Thus, increased inflammatory responses toward therapeutic IgG may paradoxically diminish the utility of the major treatment regime in this subset of CVID patients.

Polymorphism and CNV of activatory FcγRIIC is associated with increased severity of RA and ITP (106, 178). This has been attributed to expression variance in these individuals causing an imbalance between activatory and inhibitory signals.

Since the inhibitory FcγRIIB forms modulate the activation of B cells and innate effector cells, decreased expression of the FcγRIIB leads to dysregulated antibody function and increased antibody-dependant inflammatory cell responses and thus increased susceptibility to autoimmune diseases. Polymorphisms in the *FCGR2B* promoter or transmembrane domain of FcγRIIB influence receptor expression and signaling potency and are associated with susceptibility to autoimmune diseases including SLE, Goodpasture's disease, ITP, and RA (133–135, 156, 179, 180). Multiple polymorphisms in the promoter region of *FCGR2B* have been identified. The promoter haplotype *FCGR2B*−386G>C SNP in combination with *FCGR2B*-120T>A SNP (*FCGR2B*-386C +−120A) enhances promoter activity and transcription, however this enhanced haplotype has low prevalence (59, 131). *FCGR2B*-343G>C SNP is enriched in European American SLE patients and homozygous expression of *FCGR2B*-343C is linked to SLE susceptibility (131, 179). This is due to decreased AP1 transcription complex binding, which causes decreased FcγRIIB expression on B cells and macrophages and altered antigen clearance (179).

The frequency of the transmembrane polymorphism FcγRIIB-Thr^232^Ile differs among different ethnic populations, with FcγRIIB-Thr^232^ associated with SLE in Asian but not African American or European populations (134). FcγRIIB-Thr^232^ shows reduced lateral mobility in the membrane which impairs its ability to inhibit the co-localization of BCR and CD19 microclusters and consequent B cell activation (181). This causes increased B cell and myeloid cell activation (133, 136, 137), which elevates B cell (antibody) responses and heightens IgG-dependant pro-inflammatory responses, resulting in autoimmunity.

### Cancer

The roles of FcγR in cancer relate largely to the harnessing of antibody-dependant effector functions such as ADCC or ADCP by therapeutic mAbs during the treatment [reviewed in (2, 139)]. However, it also appears that mAb therapy may also have long term therapeutic benefits. Studies on DCs indicate that FcγRIIA activation is necessary and sufficient to induce a strong T cell anti-tumor cellular immunity inducing long term anti-tumor vaccine-like or “vaccinal effects” in humanized mice (48). Engagement of FcγRIIA induced DC maturation and up-regulation of costimulatory molecules, priming them for optimal antigen presentation and cross-presentation, thus stimulating long-term anti-tumor T cell memory (48).

Conversely, the inhibitory role of FcγRIIB may be disadvantageous to antibody-based therapies and other immune stimulating therapies. Thus, blocking inhibitory function of FcγRIIB on effector cells or antigen presenting cells such as DCs might be a strategy to enhance anti-tumor immune responses during immunotherapy (18, 182, 183).

## Harnessing or Targeting FcγRII for Antibody Based Therapies

Monoclonal antibodies are a versatile class of biotherapeutic drugs because of the multifunctional nature of the antibody molecule. IgG-based therapeutic mAbs are effective for the treatment of a variety of diseases due to their high specificity and affinity for their target antigen and, in some cases, their strong induction of FcγR effector functions. Depending on the nature of the disease and molecule, the mAb efficacy may depend on one or more mechanisms of action, ranging from simple antigen neutralization, complement-dependent cytotoxicity, FcγR-dependant cellular effector functions, or inhibition via FcγRIIB. Thus, effective patient responses can be dependent on FcγR based mechanisms, e.g., altered binding due to the FcγRIIA-His^131^Arg polymorphism, which influence the efficacy of therapeutic mAbs such as rituximab and cetuximab (184, 185).

The efficacy of the anti-EGFR mAb, cetuximab, and subsequent progression free survival was associated with expression of the His^131^ variant of FcγRIIA (185). Patients with the FcγRIIA-His^131^ genotype also responded better to rituximab treatment in non-Hodgkin's lymphoma (184). Conversely, FcγRIIB expression on lymphoma cells is a risk factor for anti-CD20 rituximab therapy failure due to FcγRIIB internalizing the CD20:rituximab complex and thereby reducing exposure of the opsonized lymphoma cell to the immune effector systems (186).

Inhibition of activatory FcγR could block early development of inflammatory disease. This has been explored experimentally in humanized mouse models of RA, using antibody fragments (or small molecules) designed to bind human FcγRIIA to inhibit disease (29, 187). Synthetic FcR mimetics have also been used to block the function of FcγRIIA *in vitro* (188) and the modulation of FcγRIIA and FcγRIIB function in humans (189).

FcγRIIB is a powerful modulator of ITAM-dependent receptors such as the BCR or high affinity FcεRI. Strategies to harness this powerful inhibitory capacity are being developed by engineering mAb Fc regions with enhanced and/or selective engagement with FcγRIIB. Such strategies rely on the co-engagement of FcγRIIB with the mAb-targeted activating receptor. This engineering of therapeutic mAbs with increased affinity to FcγRIIB has diverse clinical applications. Indeed, anti-CD19 binds the BCR complex and the engineered Fc co-engages FcγRIIB with increased affinity, suppressing B cell activation without B cell depletion (190, 191). This novel approach to treat autoimmune disease demonstrates the importance of understanding FcγR biology and interactions with IgG in order to optimally exploit antibody functions for specific therapies.

Another example is the anti-IgE, omalizumab, an effective treatment for allergic asthma by neutralizing IgE binding to FcεRI. Mutations introduced in XmAb7195, an omalizumab “equivalent” antibody, enhanced affinity for FcγRIIB. Like omalizumab, XmAb7195 binds to and neutralizes circulating IgE (71). However, its enhanced Fc interaction with FcγRIIB may also promote co-aggregation of FcγRIIB with the BCR of IgE+ B cells, and may suppress activation of the BCR, diminishing allergic antibody production. In addition, data from mouse studies suggest that the XmAb7195:IgE complexes are rapidly removed from the circulation via FcγRIIB expressed in the liver endothelium (71).

### FcγR Targeted Therapies

In some autoimmune diseases, auto-antibodies activate inflammatory cell effector functions against self-antigens leading to tissue destruction. One strategy used to ameliorate this destructive pathogenesis is the use of soluble FcγRs, which compete for auto-antibody binding with cell-based FcγRs thereby preventing induction of the cell-based effector functions (1, 9). Pre-clinical studies have demonstrated that the use of these soluble FcγRs suppresses the Arthus reaction, collagen-induced arthritis, and SLE (192, 193). A soluble recombinant form of FcγRIIB, named SM101, is a potential treatment for the treatment of ITP and SLE and has progressed into clinical trials (194, 195).

Small chemical entities (SCEs) specific for FcγRIIA have also been reported to inhibit immune complex-induced responses including platelet activation and aggregation, and TNF secretion by macrophages *in vitro* (187). Furthermore, *in vivo* testing of these SCEs in FcγRIIA transgenic mice also inhibited the development and stopped the progression of collagen-induced arthritis (CIA) (187). Hence, these SCE FcγRIIA antagonists demonstrated their potential as anti-inflammatory agents for pro-inflammatory immune complex-dependent autoimmune diseases.

## Conclusions

FcγRII receptors and their variants play important roles in the healthy immune response to infection, as well as in the pathologies of autoimmunity and the efficacy of therapeutic mAb treatments in cancer. Our expanding knowledge of these widely expressed FcγR and their signaling pathways may provide insight as to how we can exploit this intricate immunomodulatory system for therapeutic and diagnostic purposes. Harnessing FcR-dependent cellular effector systems through therapeutic mAbs, or by blocking effector functions, is becoming an increasingly useful tool to treat an extensive range of diseases.

## Author Contributions

JA drafted the manuscript. AC, BW, and PMH provided additional text and all authors reviewed the manuscript.

### Conflict of Interest Statement

The authors declare that the research was conducted in the absence of any commercial or financial relationships that could be construed as a potential conflict of interest.
